# Mathematical Models of Localized Muscle Fatigue: Sensitivity Analysis and Assessment of Two Occupationally-Relevant Models

**DOI:** 10.1371/journal.pone.0143872

**Published:** 2015-12-14

**Authors:** Ehsan Rashedi, Maury A. Nussbaum

**Affiliations:** 1 Department of Industrial and System Engineering, Virginia Tech, Blacksburg, Virginia, United States of America; 2 Department of Biomedical Engineering and Sciences, Virginia Tech, Blacksburg, Virginia, United States of America; Cinvestav-IPN, MEXICO

## Abstract

Muscle fatigue models (MFM) have broad potential application if they can accurately predict muscle capacity and/or endurance time during the execution of diverse tasks. As an initial step toward facilitating improved MFMs, we assessed the sensitivity of selected existing models to their inherent parameters, specifically that model the fatigue and recovery processes, and the accuracy of model predictions. These evaluations were completed for both prolonged and intermittent isometric contractions, and were based on model predictions of endurance times. Based on a recent review of the literature, four MFMs were initially chosen, from which a preliminary assessment led to two of these being considered for more comprehensive evaluation. Both models had a higher sensitivity to their fatigue parameter. Predictions of both models were also more sensitive to the alteration of their parameters in conditions involving lower to moderate levels of effort, though such conditions may be of most practical, contemporary interest or relevance. Although both models yielded accurate predictions of endurance times during prolonged contractions, their predictive ability was inferior for more complex (intermittent) conditions. When optimizing model parameters for different loading conditions, the recovery parameter showed considerably larger variability, which might be related to the inability of these MFMs in simulating the recovery process under different loading conditions. It is argued that such models may benefit in future work from improving their representation of recovery process, particularly how this process differs across loading conditions.

## Introduction

Localized muscle fatigue (LMF) is a complex phenomenon that involves reduced muscle force generation capacity and is typically associated with discomfort, pain, and a decline in desired performance. LMF can influence diverse aspects of the neuromuscular system prior to task failure (or, endurance time), and thus has been broadly defined as “any exercise-induced reduction in the ability of a muscle to generate force or power” [1,2]. The fatigue-induced reduction in muscle capacity can result from impairments in several central and/or peripheral mechanisms responsible for muscle force generation. These mechanisms are diverse, leading to substantial complexity in the fatigue process, as well as a substantial dependency of LMF on specific loading conditions [3].

LMF development and its consequences (e.g., discomfort and decline in muscle capacity), however, are important concerns in many fields such as rehabilitation, human factors engineering, and occupational health and safety. As examples of the latter, LMF has been argued as a contributing factor to the development of work-related musculoskeletal disorders [4], suggested to increase the risk for accidents such as falls [5,6], and found to compromise performance on precision tasks [7]. Again in the occupational domain, it is often of interest to quantify the presence or extent of LMF, as this can be useful for task assessment or redesign, and more generally to determine the extent to which task demands may exceed an individual’s capacity. However, it is not practical to measure LMF directly in many situations, particularly during actual task performance. As such, and given the noted dependency of LMF on loading conditions, the use of muscle fatigue models (MFMs) to predict muscle fatigue has broad potential application.

Existing MFMs has been broadly categorized into two types, *empirical* and *theoretical* [8]. Empirical MFMs are based on empirical observations and fitting to experimental data. These models are simple and suitable for some purposes (e.g., for a few or small range of task demands), though they suffer from lack of generalizability. Theoretical MFMs, on the other hand, are based on mathematical representations of physiological processes that are either presumed or supported by existing evidence. These models have utilized several approaches for predicting declines in muscle force during diverse fatiguing tasks. Some of these models are particularly relevant to task design or evaluation in occupational settings (see Table 1 of Rashedi and Nussbaum [8]), since they can be easily implemented and their underlying modeling rationale is related to voluntary contractions (and not, for example, muscle activation due to electrical stimulation).

**Table 1:** Parameter baselines, increments, and ranges used for the sensitivity analysis of two muscle fatigue models (MFM).

**MFM**	**Parameter**	**Baseline ± Increment**	**Range**
XFL [15]	F	0.01090 ± 0.00071	0.00390–0.01800
	R	0.00101 ± 0.00007	0.00032–0.00170
MCBZ [16]	F	1.06870 ± 0.04840	0.58470–1.55280
	R	0.10250 ± 0.00980	0.00500–0.20000

To improve and/or facilitate applicability of these models (such as in existing software and digital human modeling), it is useful to assess and compare the performance of these models under different loading conditions. Identifying conditions in which relatively better or worse model performance exists can serve as a basis for generating and testing formal hypotheses, which may lead to further improving such models in the future. Another useful step toward improving MFMs is to conduct a sensitivity analysis, to determine which input parameters contribute more substantially to output variability or which parameters are more influential in affecting model predictions. Such information can provide a foundation to determine where additional research is needed, for example to better specify model parameters or whether such parameters might be variables vs. constants. However, to our knowledge, outcomes of MFMs have not been compared to evaluate their consistency, nor has the noted sensitivity to model parameters been formally assessed. Such evidence is expected to facilitate more accurate predictions of muscle fatigue and recovery during complex industrial tasks, particularly in a proactive fashion. More effective proactive design, such as by integrating existing or refined MFMs in digital simulations may, in turn, reduce the development of LMF and associated adverse consequences.

## Methods

Four contemporary mathematical MFMs were initially considered, which our recent review highlighted as having the most relevance and potential application in occupational settings (i.e., predict responses to voluntary contractions, computationally efficient, and not overly complex) [8]. The first model, by Ma et al. [9] (MCBZ), consists of two first-order differential equations for fatigue development and recovery with associated constant parameters (F and R).

$$\frac{dQ(t)}{dt}=-F\;\frac{Q(t)}{MVC}\;F_{ext}(t)$$ (1A)

$$\frac{dQ(t)}{dt}=R\;(MVC-Q(t))$$ (1B)

where Q(t) is the current muscle capacity. External muscle force (F_ext_) and personal factors, such as maximum voluntary contraction (MVC) and fatigue resistance, are incorporated implicitly (Eq 1A). Fatigue and recovery are modeled separately: recovery from fatigue can only occur while a muscle is in the resting state. The recovery process is represented as in Eq 1B.

The second MFM, by Xia and Frey-Law [10] (XFL), was based on compartmental theory and divided the pool of motor units (MU) into three compartments: fatigued (M_F_), activated (M_A_), and resting MUs (M_R_). The transfer rate between compartments is proportional to compartment size, leading to three coupled, first-order differential equations. Generated muscle force is proportional to the size of active MU compartment. Similar to the MCBZ MFM, there are two constant parameters in this model (F and R, for fatigue and recovery), and C(t) is the activation-deactivation drive.

{dMRdt=−C(t)+R.MFdMAdt=C(t)−F.MAdMFdt=F.MA−R.MF (2)

The third MFM is also based on compartmental modeling, though with an additional compartment to the pool of MUs [11]. Fatigued MUs are considered to have two sub-pools of active and inactive fatigued MUs. An additional motor drive has been simulated accordingly, accounting for the transfer rates between these fatigued MUs. In practice, however, only one motor drive has been considered, which simplifies the model structure to exactly the same structure as the XFL MFM [10]. As such, no further assessment of this MFM has been undertaken here. The fourth MFM, by James and Green [12] (JG), was developed by assuming a continuum of MU twitch speed rather than having two pools of slow and fast twitch MUs. This model does not have any fatigue recovery process, however, so its performance was only evaluated here during prolonged isometric exertions.

These MFMs were implemented in Matlab 13.0 (The Mathworks, Inc. USA) using a numerical approach, which avoided the problem of analytically solving a complex set of differential equations. For the purpose of simulations, continuous differential equations in the models were transformed into discrete space using the Matlab function “C2D”. In all numerical analyses, a time step of one second was used, yielding a resolution of predicted endurance times of ± 1 sec (Matlab workspace used for generating figures is provided in S1 File).

### Loading conditions

Most previous efforts in fatigue modeling have been devoted to simulating the fatigue process, particularly using prolonged isometric contractions for modeling and validation purposes (see [8] for a review). However, this type of loading has relatively low occupational relevance, as most work tasks have intermittent resting periods. Further, prolonged and intermittent fatiguing exertions are fundamentally different, since blood flow is less of a limiting factor during the latter [13], particularly when the ratio of exertion time to rest is not large [14]. As such, a range of intermittent isometric exertions has been considered here, in additional to more simplistic, isometric contractions.

**Fig 1:**
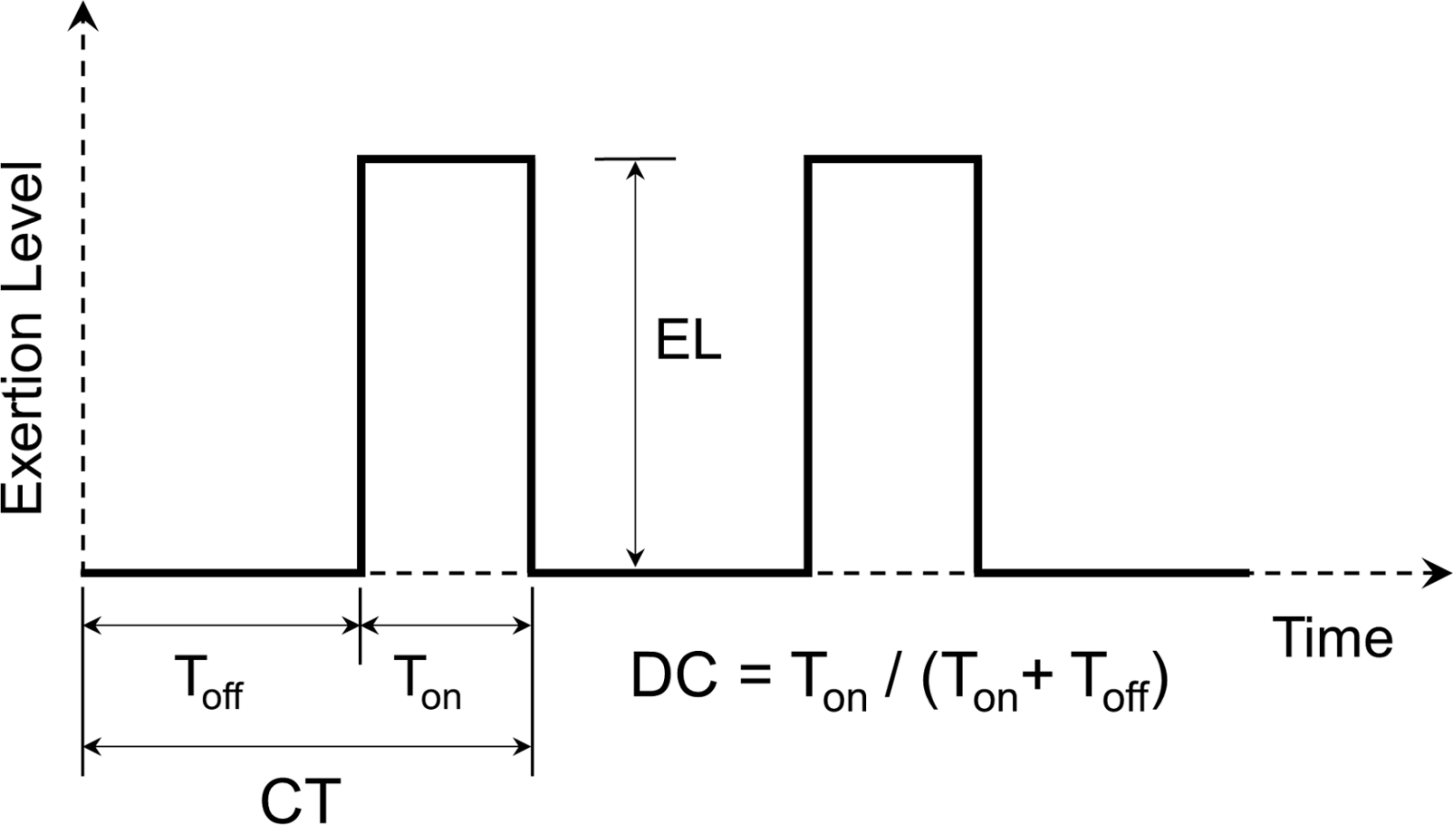
Representation of Exertion Level (EL), Duty Cycle (DC), and Cycle Time (CT). T_off_ is the rest time, and T_on_ is the portion of time when an exertion is generated.

Fatigue/recovery was simulated, as described above, using the XFL and MCBZ MFMs, for 36 different loading conditions. These conditions include all combinations of: 1) three exertion levels (EL), of 20, 40, and 60% of maximum voluntary contraction (MVC); 2) four duty cycles (DC), of 50, 65, and 80% (intermittent isometric exertions), and 100% (i.e. prolonged isometric exertion); and 3) three cycle times (CT), of 30, 60, and 240 sec (Fig 1). These specific task characteristics were chosen to correspond to a range of task parameters likely to occur in occupational settings. Other exertions, such as those involving non-isometric and/or non-isotonic contraction, were not considered due to the increase in loading and modeling complexity involved.

### Sensitivity Analysis

As noted, both the XFL and MCBZ MFMs contain two parameters representing fatigue (F) and recovery (R). The relative contributions of these parameters, or the sensitivity of model estimates to them, was determined by simultaneously varying F and R (21 values of each). Specific parameter ranges investigated, and the baseline values around which they were varied, were chosen based on previously reported values (means ± 1.5 SD) for different muscle groups and joints (Table 1) [15,16]. These ranges of parameter values were used to provide a consistent basis for assessing the models, ensuring positive values for the R parameter, and to maximize the potential for these results to be meaningful for several human muscles.

For continuous functions, partial differential techniques can be used for assessing sensitivity to one or more functional parameters [17]. In this approach, a sensitivity coefficient, Φ_i_ can be derived for a particular parameter using:

$$\Phi_{i}=(\delta Y\;/\;{\delta X}_{i})\times (X_{i}\;/\;Y)$$ (3)

where X_i_ is a model parameter, Y is the dependent variable, and the quotient X_i_ / Y is introduced for the purpose of normalization. Here, the parameters F and R are of interest, and endurance time (ET) is used as the dependent variable as a relatively direct approach for assessing predictions generated by a MFM. ET, or the time to task failure, was obtained as the time at which simulated muscle capacity failed to meet or exceed the target exertion level. Model-predicted decrements in force/torque over a fixed period of time, or rates of change over time, are other potential dependent variables. Since our purpose was to assess model sensitivity over a broad range of model parameters, utilizing constant periods of time would have been less informative, and rates of change would be challenging in cases of nonlinear responses that occur during intermittent fatigue/recovery cycles. Of note, 4 hours was used as an upper limit to ET in all analyses, given that longer durations of activity without a rest break were considered rare.

**Fig 2:**
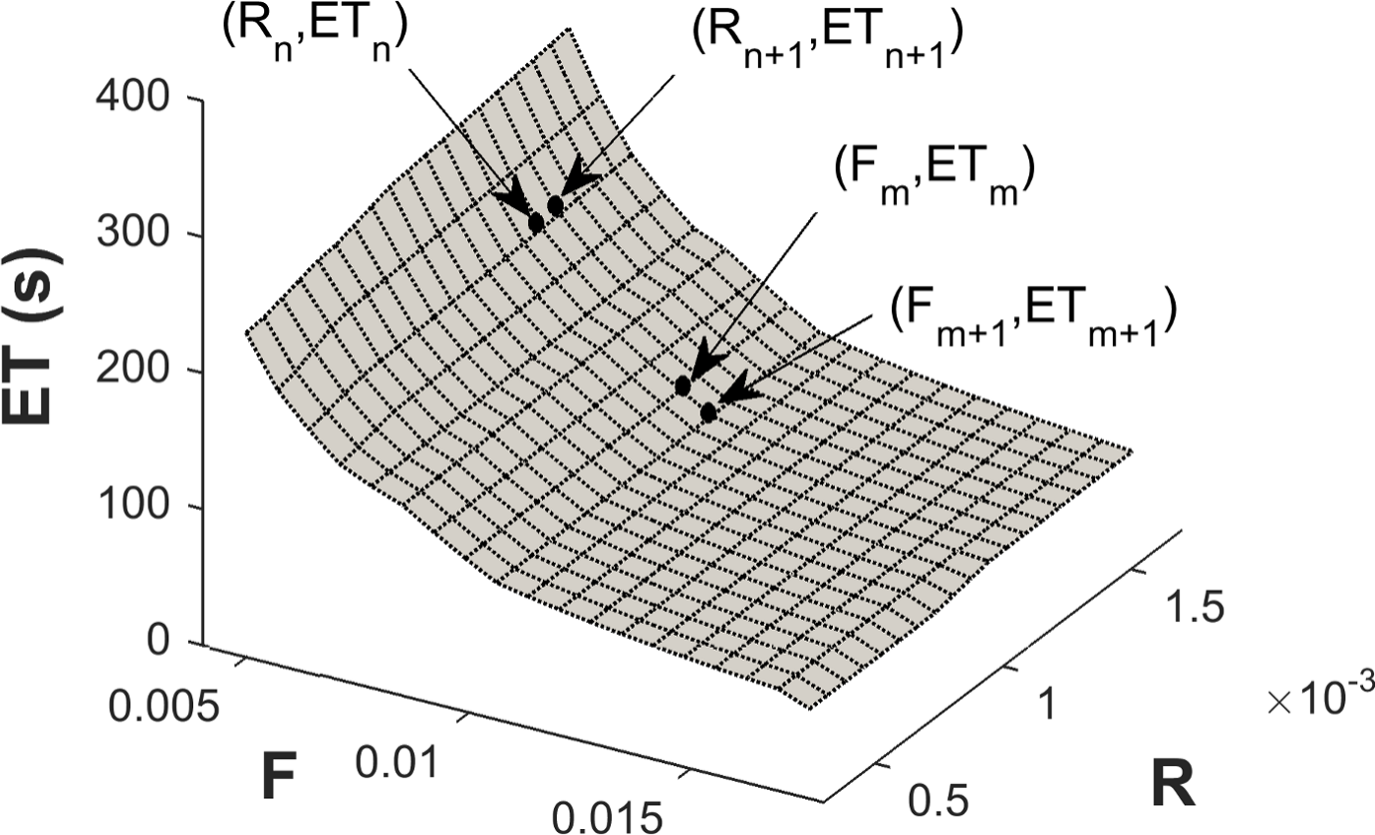
Illustration of predicted endurance times (ET) for the XFL model, in a single loading condition (EL = 0.6; DC = 65; CT = 60) and for a range of fatigue (F) and recovery (R) parameters. Methods to derive sensitivity for the F and R parameters, Φ_F_ and Φ_R_, are also illustrated, and were determined using Eqs 4 and 5.

ET, however, is not directly generated by the two MFMs (i.e., there is no analytical formulation or closed-form solution for ET), but rather is only obtained after implementing the models in a task simulation. As such, sensitivity needed to be approximated by evaluating changes in model predictions (i.e., ET) for small variations in parameters (i.e., F and R). For this, Φ_i_ was estimated as %ΔET / %ΔX_i_, or the relative change in ET for a relative change in parameter X_i_. This was determined using (see also Fig 2):

$$\Phi_{F}=[({ET}_{m+1}-{ET}_{m})*(F_{m+1}+F_{m})]\;/\;[({ET}_{m+1}+{ET}_{m})*(F_{m+1}-F_{m})]$$ (4)

$$\Phi_{R}=[({ET}_{n+1}-{ET}_{n})*(R_{n+1}+R_{n})]\;/\;[({ET}_{n+1}+{ET}_{n})*(R_{n+1}-R_{n})]$$ (5)

where *m* and *n* index over the F and R parameters, respectively.

To assess model sensitivities, ET was first determined for each of the 21*21 combinations of F and R parameters; this was done for each of the 36 noted loading conditions (Fig 2 shows an example for one loading condition). Next, ET changes with variations of the model parameters were assessed using numerical differentiation. Specifically, for each parameter (i.e., F or R), 20 sensitivity values were calculated for each of the 21 levels of the other parameter, yielding 420 sensitivities (Φ) for each parameter in a given loading condition. This yielded a total of 420 x 36 = 15,150 sensitivity parameters for each of F and R, and in each model.

**Fig 3:**
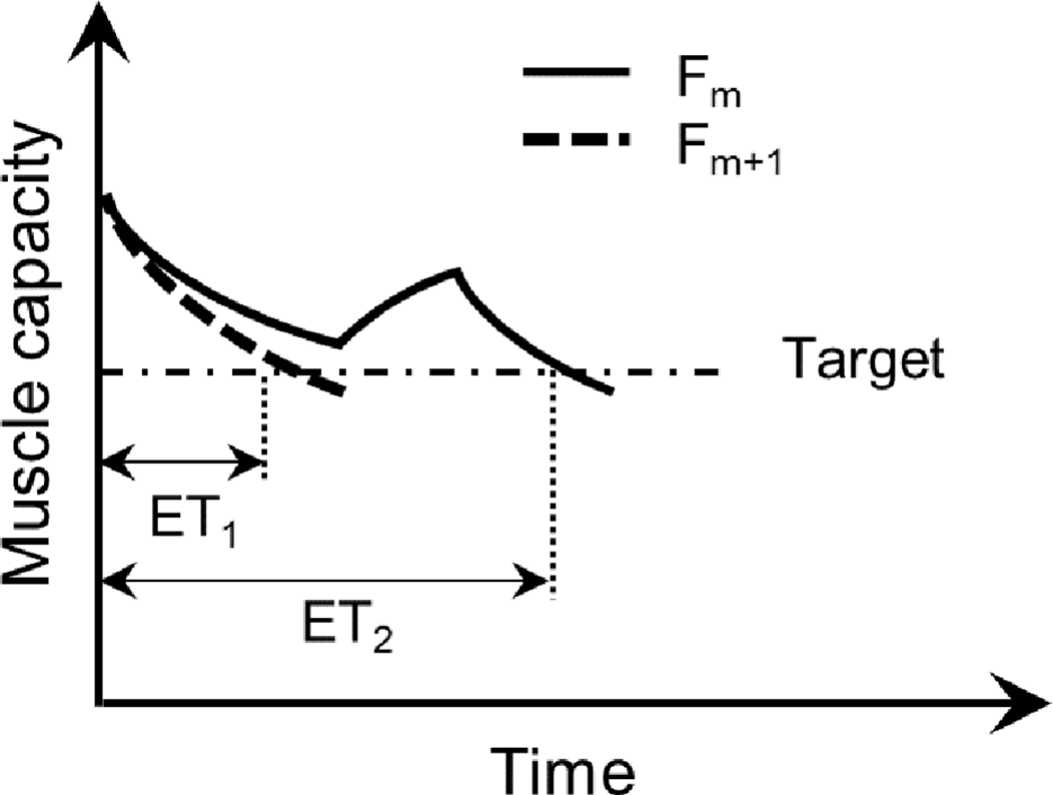
Representation of a considerable change in endurance time (ET) that can occur due to relatively small alterations in MFM parameters. Here, ET_1_ is substantially shorter than ET_2_ despite a small increment in F (from F_m_ to F_m+1_).

**Fig 4:**
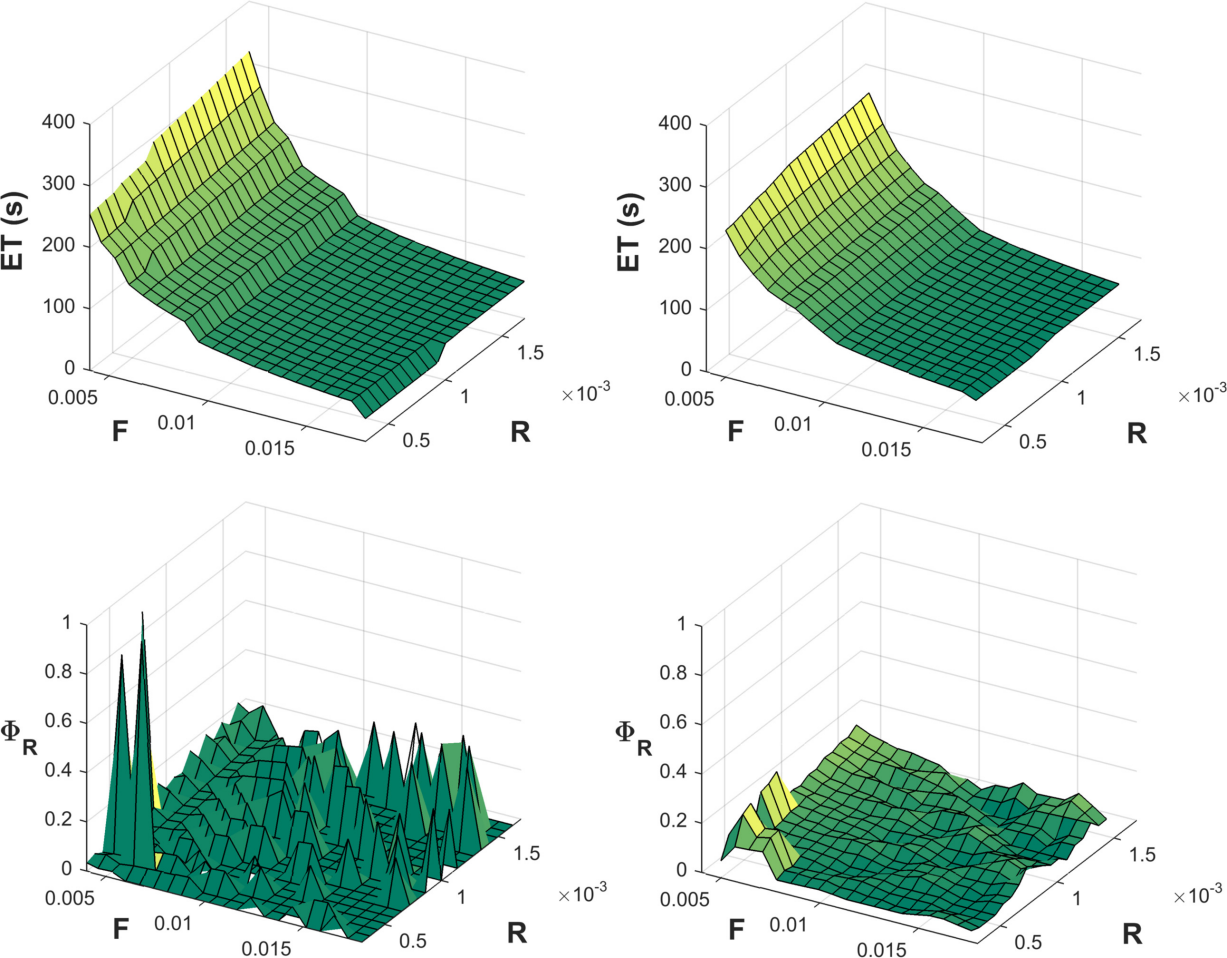
Example illustration of the effects of 2D smoothing of endurance time (ET) predictions and the derived recovery sensitivity parameter (Φ_R_). Original values of both are depicted in the left figures, and after smoothing on the right. For this illustration, the same model and loading condition was used as in Fig 2.

Preliminary results demonstrated large “spikes” in calculated sensitivity parameters, and which were clearly due to inherent substantial changes in ETs with relatively small changes in parameters during intermittent loading. For example, small alterations in either the F or R parameters can lead to one more or less exertion cycle that can be completed, and in some cases this would represent a considerable relative change in ET (Fig 3). Such substantial ET changes will, in turn, lead to large changes in sensitivity parameters given the differentiation involved. To avoid this, and to better reflect the general patterns of sensitivity, ET values were first “smoothed”. This smoothing was done using a mean filter over a rectangle of size 3*3 (i.e., replacing each ET by the mean of itself and all neighboring points), Sensitivity parameters are not reported on the boundaries of the matrix of F and R parameters, both to avoid edge effects and since a full rectangle was not possible. Fig 4 illustrates an example of this smoothing procedure and associated effects on the derived recovery sensitivity parameter.

Subsequently, sensitivity parameters were obtained as a function of task parameters (i.e., EL, DC, and CT). For this purpose, the mean sensitivity of one parameter was derived for the midrange value of the other parameter: for each specific combination of task parameters, the mean of eight Φ_F_ values was obtained while setting the R parameter to its midrange value. This approach was used to demonstrate the overall trends regarding model sensitivity to the F and R parameters across different loading conditions.

### MFM Comparisons

To compare the MCBZ [9] and XFL [10] MFMs, their ability to predict ETs was assessed for three types of loading conditions, and for which empirical data (ETs) was available from the literature for a consistent muscle group. Specific loading types assessed were: 1) prolonged isometric exertions; 2) intermittent isometric exertions; and, 3) a combination of these two load types. For each loading type, individual model parameters (F and R) were obtained that minimized deviations between model predictions of ETs and existing empirical data, with such error quantified as the root mean square deviation (RMSD). For prolonged isometric loading, the intensity-ET relationship presented by Frey Law and Avin [18]was used. For intermittent isometric contractions, four empirical studies were available that each involved the hand/grip (Table 2). Parameter values for both models were then obtained using an iterative search (over a larger range comparing to Table 1), to identify the combination of F and R that produced the least RMSD error. For prolonged isometric contractions, RMSD was minimized across 19 exertion levels (0.1% - 100% MVC, in 5% increments). For intermittent isometric exertions, RMSD was minimized across the four conditions available (Table 2). For the combination, RMSD was minimized for the total of 23 conditions (19 isometric + 4 intermittent). Since the iterative search for parameters was done separately for the three loading, three values of RMSD for each model were obtained.

**Table 2:** Empirical studies involving hand/grip exertions that included intermittent isometric loading conditions with reported mean (SD) endurance times (ET).

**Study**	**EL (% MVC)**	**DC (%)**	**CT (sec)**	**ET (sec)**
Carpentier et al. [19]	50	65	20	502 (213)
Fujimoto and Nishizono [20]	40	60	10	720 (240)
Fulco et al. [21]	50	50	10	444 (48)
Pitcher and Miles [22]	80	50	10	62 (16)

## Results

### Prolonged Isometric Contractions

**Fig 5:**
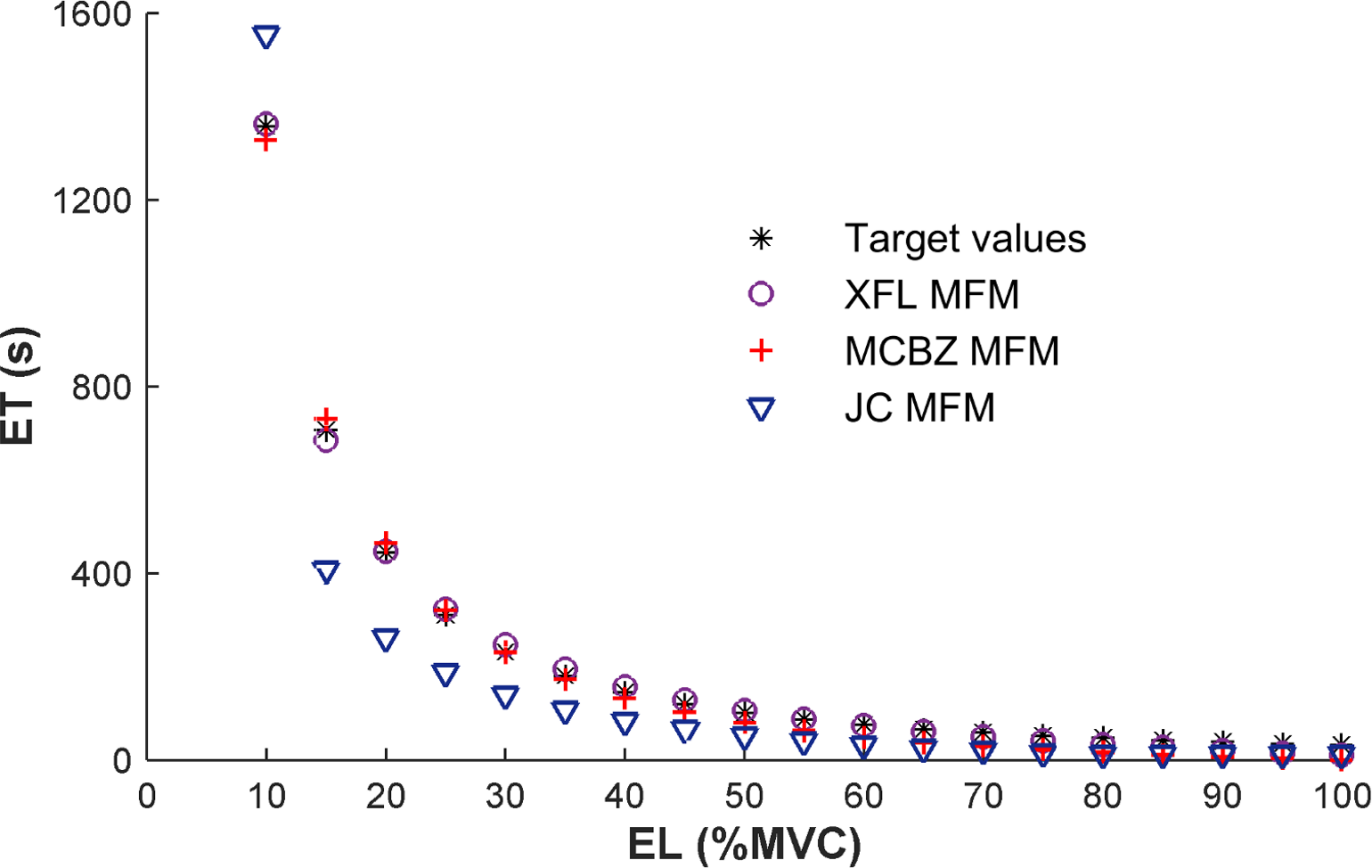
Endurance Time (ET) predictions of three MFMs for sustained isometric exertions. Target values are from data reported by Frey Law and Avin [18].

ET predictions from the XFL, MCBZ, and JC MFMs during prolonged isometric contractions are depicted in Fig 5 (see Table 3 for optimized parameter values for the former two MFMs). Notably, in MCBZ MFM, recovery process is only active during true rest, thus, R parameter during prolonged isometric contractions is not applicable for this MFM. Both the XFL and MCBZ MFMs demonstrated substantial consistency with reported ET values [18], and with R^2^ >0.99 for both. The JC MFM output was less consistent with these target values (R^2^ ~ 0.91), and overpredicted and underpredicted ETs for low and moderate effort levels, respectively. As noted earlier, the JC MFM was not considered further (i.e., for intermittent contractions), since this model does not include a recovery process.

**Table 3:** Optimized MFM parameters obtained for prolonged and intermittent isometric exertions, and the combination of both.

MFM	Prolonged Exertions			Intermittent Exertions			Combined Exertions		
	F	R	RMSD (s)	F	R	RMSD (s)	F	R	RMSD (s)
XFL	0.0108	0.0008	14.0	0.0162	0.0065	206.1	0.0143	0.0014	292.3
MCBZ	1.0400	NA*	24.7	0.8428	0.1930	217.3	0.9074	0.0982	265.6

* NA: not applicable

### Sensitivity Analysis

**Fig 6:**
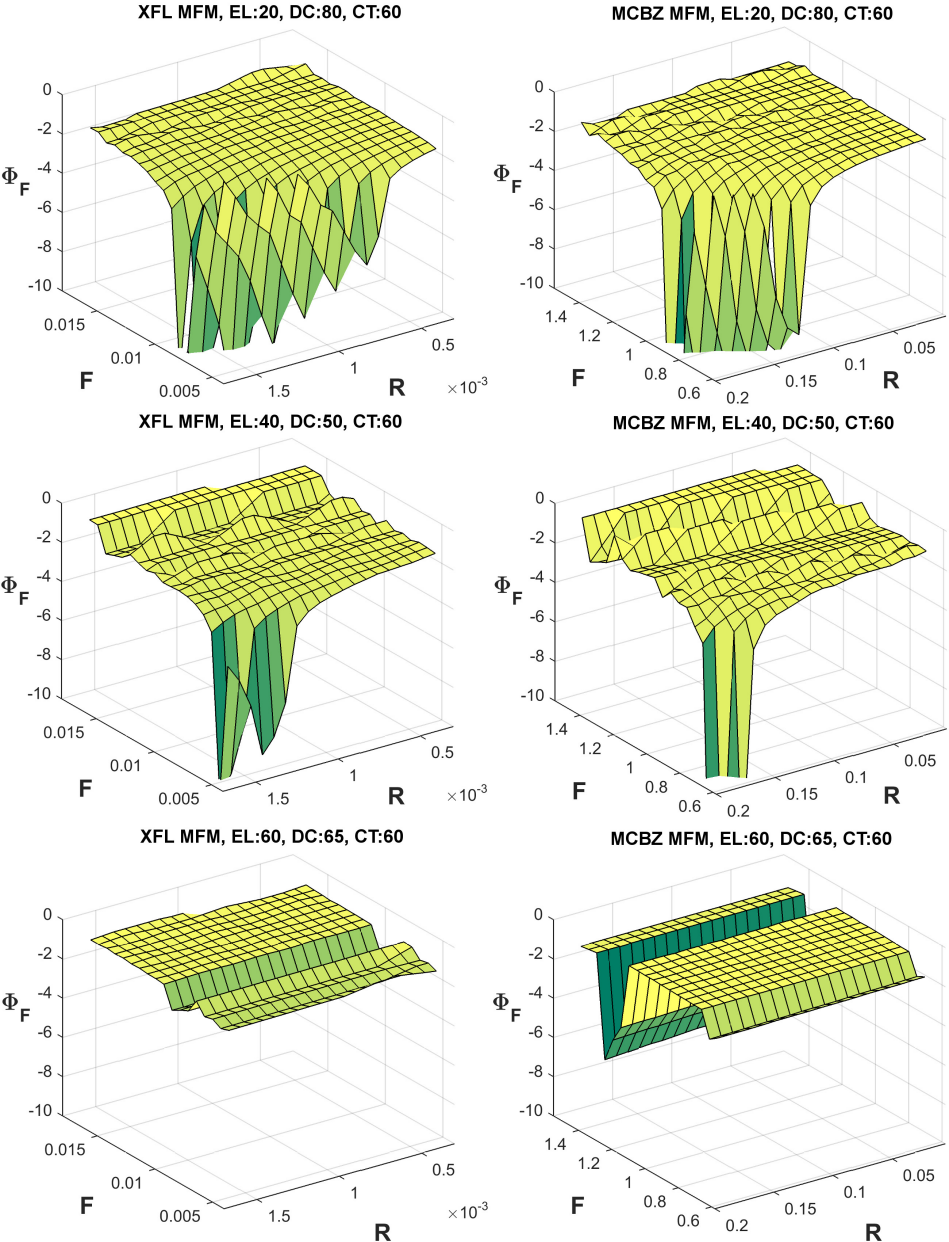
Representative examples of fatigue sensitivity parameters (i.e., Φ_F_) for the XFL (left) and MCBZ (right) MFMs. Φ_F_ values were determined using Eq 4, iterating the F and R parameters over a wide range (Table 1). Higher values of Φ_F_ indicate larger relative sensitivity to changes in F values. Some Φ_F_ values (>10) are not shown, to better illustrate patterns of responses.

**Fig 7:**
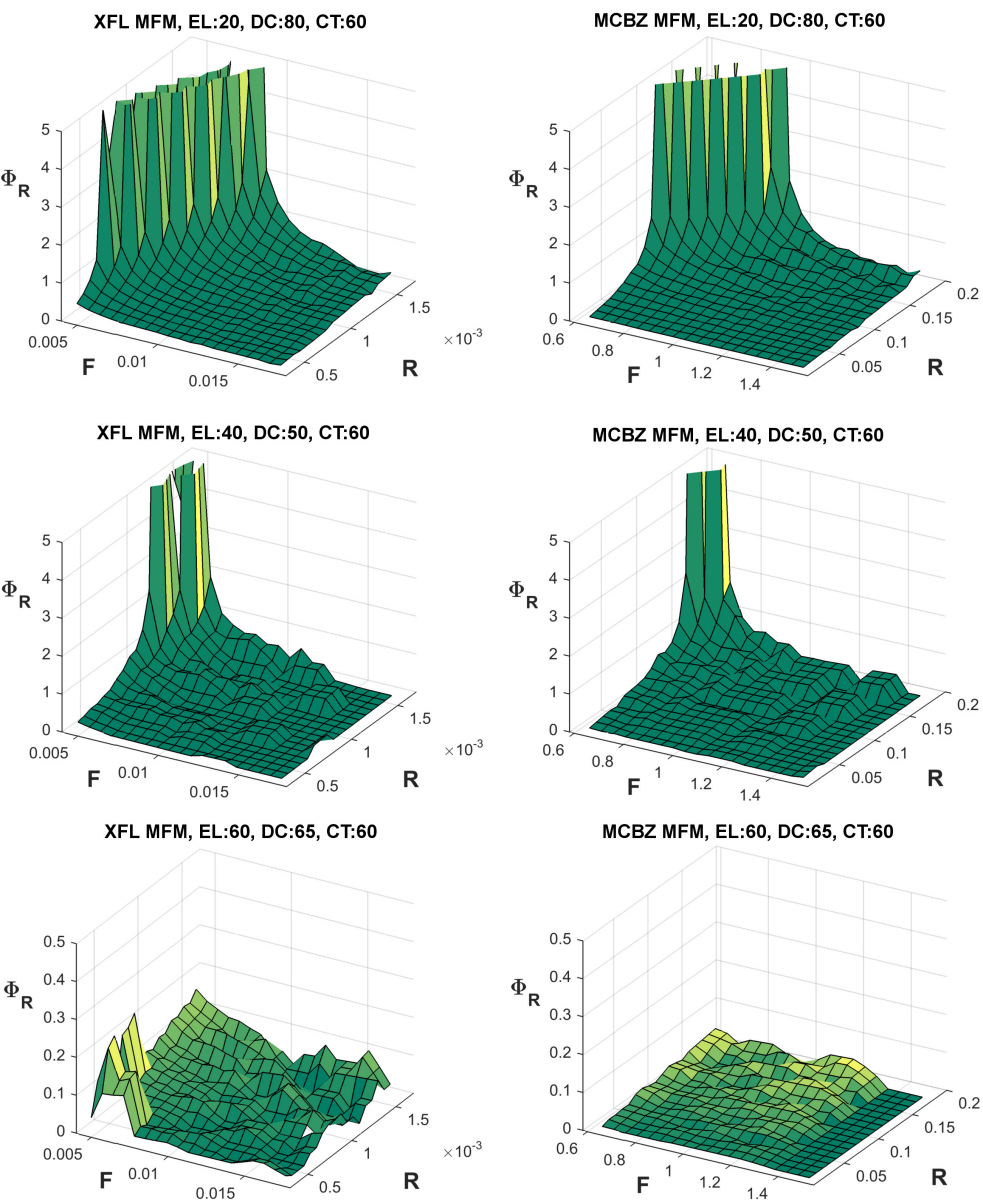
Representative examples of recovery sensitivity parameters (i.e., Φ_R_) for the XFL (left) and MCBZ (right) MFMs. Φ_R_ values were determined using Eq 5, iterating the F and R parameters over a wide range (Table 1). Higher values of Φ_R_ indicate larger relative sensitivity to changes in R values. Some Φ_R_ values (>5) are not shown, to better illustrate patterns of responses.

Representative examples of fatigue and recovery sensitivity parameters for both the XFL and MCBZ MFMs are presented in Figs 6 and 7, respectively. Of note, all Φ_F_ values are negative, since a larger F parameter leads to smaller ET, and all Φ_R_ values are positive, since an increment in R results in a larger ET. In general, and except for some localized steep changes related to the inherent characteristics of intermittent exertions noted above (*cf* Fig 3), Φ_F_ was relatively “flat” for higher exertion levels. The maximum magnitude of Φ_F_ was also larger than Φ_R_ in both MFMs. For lower exertion levels, both models were more sensitive to changes of the F parameter (i.e., larger Φ_F_) at lower F values and at higher R values. Similarly, both models were more sensitive to R parameter changes (i.e., larger Φ_R_) at lower F values and higher R values. Such conditions, with lower F and higher R, are those involving longer ETs. As such, sensitivity to both parameters, in both models, was highest for those tasks with the longer ETs.

**Fig 8:**
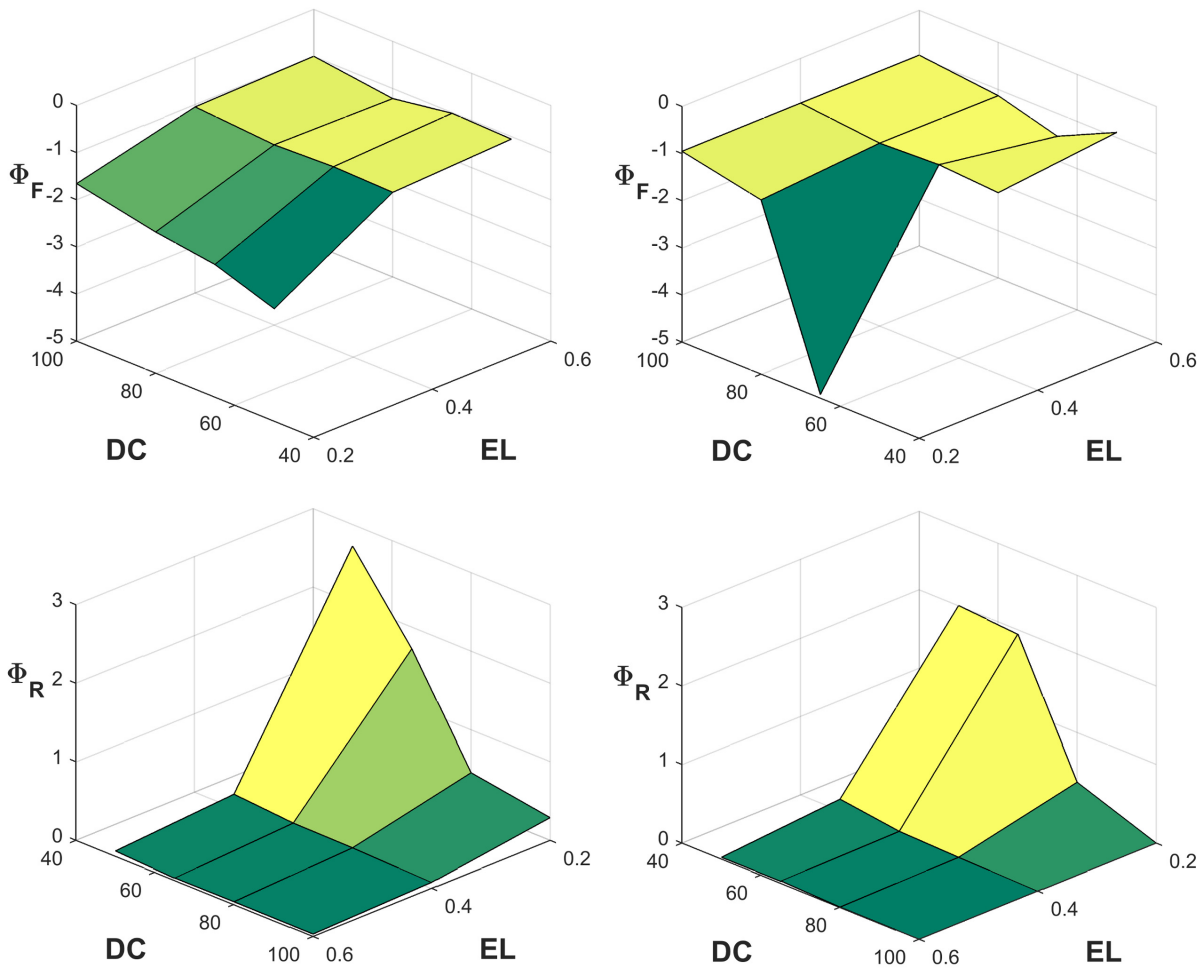
Sensitivity values of one model parameter (F and R) at the midrange of the other parameter, for different values of task parameters in the XFL (left) and MCBZ (right) MFMs (CT = 60 s). Note that in this figure the viewpoint is different for Φ_F_ and Φ_R_, to better visualize the patterns of responses.

Mean sensitivity parameters were obtained as a function of task parameters (i.e., EL, DC, and CT), with representative examples shown in Fig 8 (similar results were obtained for the remaining CTs). Both MFMs were more sensitive to alterations of their parameters at lower ELs and DCs, which are conditions involving lower physical demands. Consistent for all CTs, both Φ_F_ and Φ_R_ were largest for the lower DC (50%) and lowest EL (20% MVC). More generally, Φ_F_ and Φ_R_ were both larger for lower DCs and ELs.

### MFM Comparisons

The XFL and MCBZ MFMs were used to predict ET during three loading types (prolonged, intermittent, and combined), after obtaining optimized parameters for each type. Resulting parameters and associated RMSD values are presented in Table 3. The MCBZ MFM yielded a lower correspondence (higher RMSD) between predicted and reported ETs in prolonged and combination loading types. For the XFL MFM, the F and R parameters increased between prolonged and intermittent exertions by ~50 and 700%, respectively. For the MCBZ MFM, there was a ~20% decrease in the F parameter. After fitting the models simultaneously to combined prolonged and intermittent loading conditions, optimized F and R parameter values were between respective values obtained for the two separate loading types. Similarly, RMSD values for both MFMs were largest when fitting to the combined vs. separate loading types.

## Discussion

Localized muscle fatigue (LMF) is a complex phenomenon, given the diverse mechanisms that underlie both initiation of and recovery from fatigue. There is substantial value in quantifying LMF, however, since it has potential adverse effects on both performance and injury risk. Given the number of task-related variables (as well as intra- and inter-individual differences) that impact LMF, it is not practical to measure LMF for all possible conditions. Nor is this practical (or often feasible) in the workplace, specifically measuring fatigue for each worker. Therefore, it of interest and potential practical utility to predict LMF development and/or endurance capability using MFMs, given a set of task demands and without the need for direct measurements of LMF (e.g., from an actual worker or using a mock-up). MFMs have been broadly categorized into two types, *empirical* and *theoretical* [8]. We focused here on *theoretical* MFMs, because of their broader range of utility, in contract to empirical MFMs that appear mainly useful for a narrower set of specific applications. Based on a previous review of the literature [8], four MFMs were initially chosen, from which a preliminary assessment led to two of these (XFL and MCBZ) being considered for comprehensive analysis. Specifically, this analysis involved assessing the sensitivity of model predictions to alteration of their parameters, and a comparison of the models’ ability to predict ET in different loading conditions.

The sensitivity analysis revealed that both models had higher (normalized) sensitivity to the F parameter, suggesting the dominance of the fatigue vs. recovery parameters. Except for some relatively large but localized changes, related to inherent characteristics of intermittent exertions (Fig 3), sensitivity parameters were relatively “flat” over the ranges investigated, particularly for higher levels of EL and DC. Both Φ_F_ and Φ_R_ demonstrated larger values at lower values of F and higher values of R (Figs 6 and 7). Since lower F and higher R values yield lower rates of fatigue development over time, and thus longer endurance times, both MFMs appear to be more sensitive to their parameters in less demanding loading conditions. This same outcome was observed from averaged values of sensitivity parameters across different task conditions (Fig 8), in which both models were more sensitive at lower values of EL and DC. Lower EL and DC again indicate less demanding loading conditions, in which the fatigue process would be relatively less active compared to the more active recovery process. A larger sensitivity of models for “easier” tasks may exacerbate the challenges in predicting LMF for such tasks. In the occupational domain, MFMs are probably most useful at predicting LMF for these lower demanding tasks, since, compared to more physically demanding conditions, such tasks are less often the target of task analyses and redesign to reduce or eliminate the hazardous exposure, for example through automation or use of assistive devices [23,24].

Regarding their ability to predict ET during prolonged isometric conditions, both the XFL and MCBZ MFMs were able to nearly duplicate empirical intensity-ET values [18]. During more complex intermittent contractions, the optimized F parameter in the XFL MFM [10] increased ~50%, while the R increment was substantially larger (i.e., ~700%) (Table 3). Relatively smaller alterations of the F parameter between prolonged and intermittent contractions might suggest that a comparable fatigue process is involved in both contraction types, such as due to similar excitation-contraction processes. In fact, Xia [25] assumed such a similarity in developing his recently modified MFM (discussed below). In contrast to the fatigue process, however, fundamental differences exist in the recovery process between prolonged and intermittent contractions. During rest periods in intermittent contractions, blood flow increases [26], resulting in muscle reperfusion [27]. Faster removal of metabolites (e.g., lactic acid from prior muscle fatigue) in a complete rest condition may expedite the recovery process, and can account for the higher values of the R parameter predicted for intermittent vs. prolonged contractions. Furthermore, in prolonged contractions and depending on the intensity of exertion, blood flow occlusion may prevent the removal of fatigue byproducts and replacement of oxygen and glucose in muscle [28]. These evidence justify the observation of substantially different recovery process between pronged and intermittent exertions.

To further explore the performance of these models in different loading conditions, additional assessments were completed. Optimized parameters for both MFMs were first obtained for prolonged contractions (Table 3), and these parameters were subsequently used to predict ET in intermittent contractions. From this, both models under-predicted ETs for intermittent loading conditions (by ~80–125%). Such under-prediction may have resulted from overestimating the rate of fatigue and/or underestimating the rate of recovery. Result of optimizing the MFM parameters for intermittent contractions (Table 3), showed the latter speculation might be more likely. In other words, a deficiency in simulating the recovery process is more probable, since the R parameter in XFL MFM demonstrated much larger changes between the two conditions (i.e., ~700%). As such, the recovery parameter may need to be distinct between–or specified as a function of–different loading conditions. A fundamentally new approach to simulating the recovery process may also be needed.

To address the limitations of the XFL MFM in accurately predicting changes in muscle capacity during more complex loading conditions (e.g., intermittent contractions), recent studies [25,29,30] have introduced new approaches to simulate the recovery process [10]. Rather than assuming a constant R parameter, Xia [25] proposed having R vary based on the exertion level to reflect changes in blood flow in muscle recovery. However, outcomes with this modification were not substantially different, possibly due to over-simplification of the relationship between blood flow and muscle contraction. Looft [29] introduced a multiplier to the model, specifically to increase the rate of recovery in rest periods (reflecting post-contraction reperfusion). He fit the XFL MFM with the new rest multiplier to empirical data from the literature and reported improvements in predicting muscle fatigue during intermittent contractions. However, substantially larger errors were reported after assessing the performance of this modified version of the model for predicting ETs [29]. As such, it was concluded that using the rest multiplier may not be suitable for predicting ETs during intermittent contractions.

More recently, Sonne and Potvin [30] sought to increase the biological fidelity of the original XFL MFM [10], by modifying recovery and fatigue rates to represent graded physiological MU characteristics. Outcomes of this new modeling approach were compared with those from the original model in two conditions: 1) with original parameter values, and 2) with optimized parameter values for intermittent contractions. These authors demonstrated that, while the modified model can provide better predictions of muscle fatigue than the model with original parameter values, it has similar performance to the original model with parameter values optimized for new experimental conditions. Meanwhile, the new model did not provide good estimations of ET for prolonged isometric contraction, particularly at lower exertion levels (<40% MVC). While these alternative modeling approaches have demonstrated the potential for improved predictions in more complex intermittent contractions, performance was actually compromised for simpler loading conditions (i.e., prolonged isometric contractions). Such outcomes are similar to what was found here, specifically that model predictions are ineffective when done simultaneously for both prolonged and intermittent contractions (i.e., the largest errors were found for mixed loading conditions, a combination of prolonged and intermittent contractions).

MFM can facilitate the prediction of LMF development and/or endurance capability, providing potential benefits for tasks analysis and pro-active assessment and potentially obviating the need for the direct measurements of LMF. Two MFMs with practical utility for application in occupational settings were assessed here, in the context of prolonged and intermittent contractions. As this work examined only static exertions, future studies would benefit from incorporating dynamic contractions, which are more complex due to inherently larger alterations in MU recruitment and blood flow. Another limitation of the current study was the focus on only one muscle group (i.e., hand/grip muscles). Subsequent work should assess MFM performance for other muscle groups, since fatigue and recovery processes are not only task dependent, but also dependent on the muscle group involved (e.g., related to differences in fiber type distribution). Of note, the four studies from which data were used to assess model performance (Table 2) did not target consistent muscles group. Specifically, the studies address the first dorsal interosseous, adductor pollicis, and “grip” muscles. Along with typical inter-individual variability, which can be substantial, this increases the variability in ET, given differences in fiber type distributions of the involved muscles. Future work evaluating and comparing MFMs should ideally use more comparable data sets. Similarly, MFMs should be evaluated to assess their potential to account for important inter-individual differences, such as related to gender and aging.

In summary, two MFMs were assessed here in terms of their sensitivity to inherent model parameters and their ability to predict ET in both prolonged and intermittent exertions. The two MFMs were, in general, more sensitive to the alterations of the F parameter that represents the rate of muscle fatigue. Both models demonstrated a higher sensitivity to their F and R parameters in conditions involving lower to moderate levels of effort, though such conditions maybe those that are of most practical interest in the occupational domain. The ability of these models ability to predict ET was inferior for mixed loading condition (a combination of prolonged and intermittent contractions). When optimizing model parameters for different loading conditions, the R parameter showed considerably larger variability, which might be related to the inability of these MFMs in simulating the recovery process under different loading conditions. For future application, improved model predictions of fatigue and recovery are needed, especially across diverse loading conditions, and a specific focus on an improved representation of recovery processes is recommended.

## Supporting Information

S1 File Matlab workspace including all variables used for generating figures. (MAT)

## Abbreviations

CT: Cycle time
DC: Duty cycle
EL: Exertion level
ET: Endurance time
F: Fatigue parameter
JC: James et al. (2012) MFM
LMF: Localized muscle fatigue
MCBZ: Ma et al. (2009) MFM
MFM: Muscle fatigue model
MU: Motor unit
MVC: Maximum voluntary contraction
R: Recovery parameter
RMSD: Root mean square deviation
XFL: Xia and Frey Law (2008) MFM
